# Axon Guidance of Sympathetic Neurons to Cardiomyocytes by Glial Cell Line-Derived Neurotrophic Factor (GDNF)

**DOI:** 10.1371/journal.pone.0065202

**Published:** 2013-07-03

**Authors:** Keiko Miwa, Jong-Kook Lee, Yoshiko Takagishi, Tobias Opthof, Xianming Fu, Masumi Hirabayashi, Kazuhiko Watabe, Yasuhiko Jimbo, Itsuo Kodama, Issei Komuro

**Affiliations:** 1 Department of Cardiovascular Research, Research Institute of Environmental Medicine, Nagoya University, Nagoya, Japan; 2 Department of Genetics, Research Institute of Environmental Medicine, Nagoya University, Nagoya, Japan; 3 Departments of Medical Laboratory Science, Faculty of Health Sciences, Hokkaido University, Sapporo, Japan; 4 Department of Cardiovascular Regenerative Medicine, Osaka University Graduate School of Medicine, Suita, Japan; 5 Department of Cardiovascular Medicine, Osaka University Graduate School of Medicine, Suita, Japan; 6 Experimental Cardiology Group, Center for Heart Failure Research, Academic Medical Center, Amsterdam, The Netherlands; 7 Department of Medical Physiology, University Medical Center Utrecht, Utrecht, The Netherlands; 8 Department of Cardiovascular Surgery, Graduate School of Medicine, Nagoya University, Nagoya, Japan; 9 Center for Genetic Analysis of Behavior, National Institute for Physiological Sciences, Okazaki, Japan; 10 Laboratory for Neurodegenerative Pathology ALS, Tokyo Metropolitan Institute of Medical Science, Tokyo, Japan; 11 Graduate School of Frontier Sciences, University of Tokyo, Kashiwa, Japan; 12 Department of Cardiovascular Medicine, Graduate School of Medicine, University of Tokyo, Tokyo, Japan; Osaka University Graduate School of Medicine, Japan

## Abstract

Molecular signaling of cardiac autonomic innervation is an unresolved issue. Here, we show that glial cell line-derived neurotrophic factor (GDNF) promotes cardiac sympathetic innervation in vitro and in vivo. *In vitro*, ventricular myocytes (VMs) and sympathetic neurons (SNs) isolated from neonatal rat ventricles and superior cervical ganglia were cultured at a close distance. Then, morphological and functional coupling between SNs and VMs was assessed in response to GDNF (10 ng/ml) or nerve growth factor (50 ng/ml). As a result, fractions of neurofilament-M-positive axons and synapsin-I-positive area over the surface of VMs were markedly increased with GDNF by 9-fold and 25-fold, respectively, compared to control without neurotrophic factors. Pre- and post-synaptic stimulation of β_1_-adrenergic receptors (BAR) with nicotine and noradrenaline, respectively, resulted in an increase of the spontaneous beating rate of VMs co-cultured with SNs in the presence of GDNF. GDNF overexpressing VMs by adenovirus vector (AdGDNF-VMs) attracted more axons from SNs compared with mock-transfected VMs. *In vivo*, axon outgrowth toward the denervated myocardium in adult rat hearts after cryoinjury was also enhanced significantly by adenovirus-mediated GDNF overexpression. GDNF acts as a potent chemoattractant for sympathetic innervation of ventricular myocytes, and is a promising molecular target for regulation of cardiac function in diseased hearts.

## Introduction

Sympathetic nervous system plays a critical role in regulating cardiac function in balance with parasympathetic activity. However, in diseased hearts, nonuniform myocardial innervation in association with enhanced sympathetic activity is supposed to create a substrate for life-threatening arrhythmias [1]; [2]. The abnormal sympathetic innervation has been caused by “nerve sprouting” of sympathetic origin and “disordered” reinnervation in pathological condition such as myocardial infarction or heart transplantation[3]–[5]. Thus, it is important to understand how cardiac cells attract sympathetic axons in orderly manner.

A plethora of neurotrophic factors is involved in sympathetic innervation and nerve sprouting. Several experimental studies have shown that nerve growth factor (NGF) facilitates sympathetic innervation [6]–[8]. Recently, glial cell line-derived neurotrophic factor (GDNF) was shown to be upregulated in rats after chemically induced sympathectomy, suggesting its role in sympathetic nerve regeneration [9]. Upregulation of both NGF and GDNF was also demonstrated in rats with *Trypanozoma cruzi* infection (Chagas disease) causing sympathetic as well as parasympathetic denervation [10]. Artemin, a neurotrophic factor of the GDNF family, was shown to promote the development of sympathetic innervation along blood vessels by enhancing sympathetic axon growth [11]; [12]. We have previously reported that in the rat model, GDNF enhances sympathetic axon growth toward cardiomyocytes. Under pathophysiological conditions, the effect of GDNF was more potent than that of the neurotrophic factors, such as NGF or brain-derived neurotrophic factor (BDNF) or ciliary neurotrophic factor (CNTF) [13].

Here, we present that *in vitro* proximity co-cultures of rat neonatal ventricular myocytes (VMs) and sympathetic neurons (SNs) obtained from the superior cervical ganglia showed: (i) GDNF is more potent than NGF in stimulating sympathetic axon growth and enhancing functional coupling between SNs and VMs; (ii) using VMs overexpressing GDNF (AdGDNF-VMs), we have shown a potent action of endogeneous GDNF for sympathetic “axon guidance”. Using the adult rat hearts *in vivo*, we also demonstrate that: (iii) axon outgrowth toward the denervated myocardium after cryoinjury is enhanced by GDNF overexpression. GDNF may become a novel target in restoring impaired sympathetic innervation of the diseased heart.

## Materials and Methods

All procedures were conducted in accordance with a protocol approved by the Animal Experimentation Committee, Research Institute of Environmental Medicine, Nagoya University.

### Cultures of Ventricular Myocytes and Sympathetic Neurons

**Figure 1:**
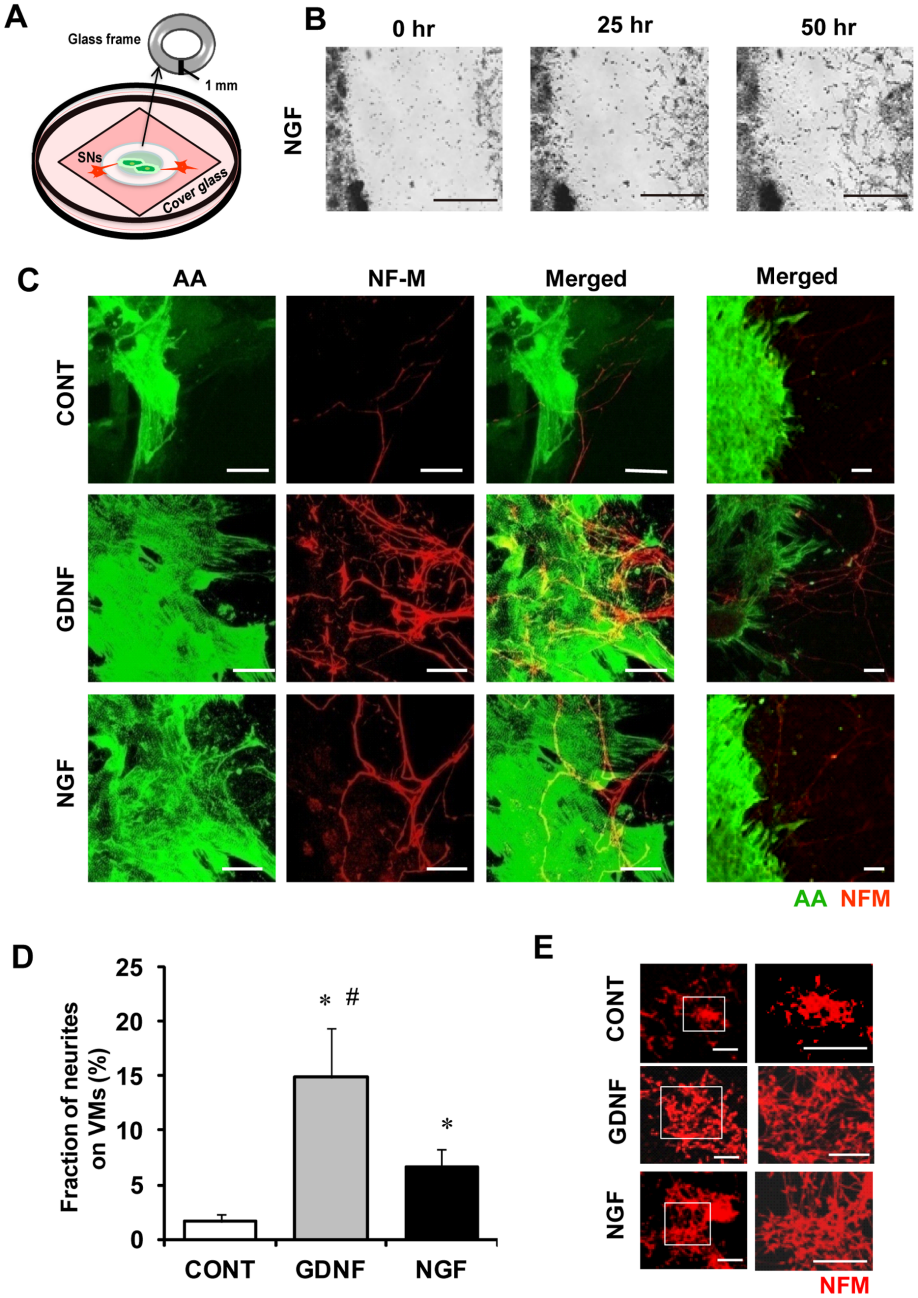
Effects of GDNF and NGF on axon outgrowth of sympathetic neurons toward cardiomyocytes. Proximity co-cultures of SNs/VMs were treated for 5 days with either GDNF (10 ng/ml) or NGF (50 ng/ml), and immunolabeled for α-actinin (AA; green) and neurofilament-M (NFM; red). Co-cultures treated with vehicle only were used as control (CONT). **A)** A schema of proximity co-culture: SNs and VMs were separately seeded inside or outside of a glass ring. Then, the glass ring was removed for observation. **B)** Representative time-lapsed images of axons growth of SNs (right) toward VMs (left) with NGF (50 ng/ml). Sequential images are available in Supporting video S1. Bars indicate 500 µm. **C)** Representative fluorescence images (left: AA, middle: NF-M, right: merged). The axon projection toward VMs in the culture treated with GDNF (middle) or NGF (bottom) is much more abundant than CONT (top). Bars indicate 20 µm. **D)** The fraction of axons (NFM-positive area) distributing on VMs (AA-positive area). Values are means±standard deviation (SD) of 10 co-cultures in each group. *Significantly different from CONT at p<0.05. #Significantly different from co-cultures treated with NGF at p<0.05. **E)** Representative fluorescence images of mono-culture of SNs with or without neurotrophins (NGF or GDMF). Bars indicate 200 µm.

**Figure 2:**
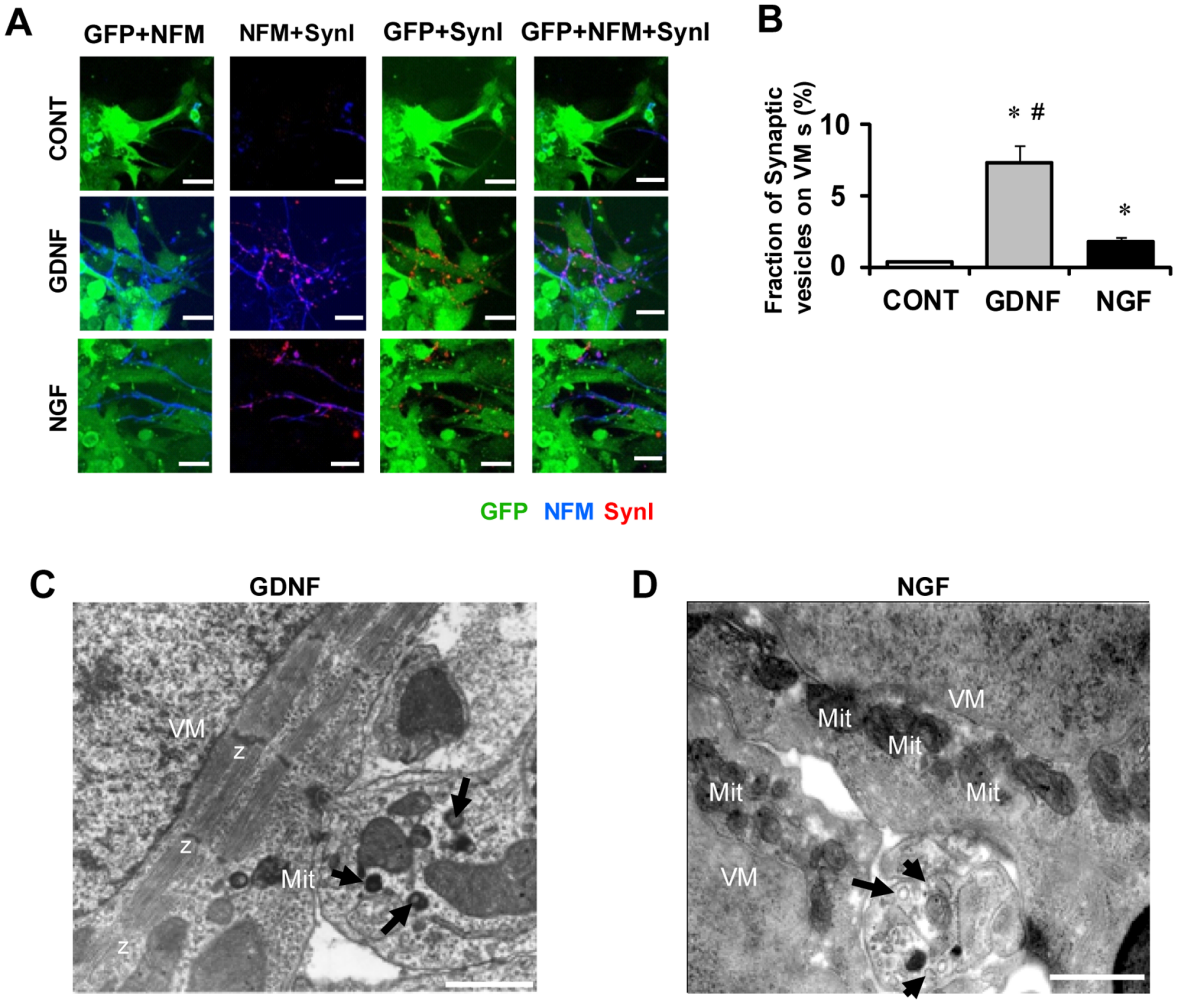
Synapse formation between cardiac cells and sympathetic neurons and ultrastructure of junctions between VMs and SNs. Proximity co-cultures of GFP-expressing VMs/SN were immunolabeled for neurofilament-M (NFM, blue) and synapsin I (SynI, red) to visualize synapse formation between SNs and VMs. **A)** Representative fluorescence images of co-cultures treated with vehicle only used as controls (CONT, top), co-cultures treated with GDNF 10 ng/ml for 5 days (middle), and treated with NGF 50 ng/ml for 5 days (bottom). Pictures from left to right are labeled for GFP+NFM, NFM+SynI, GFP+SynI, and GFP+NFM+SynI, respectively. The synaptic vesicles are clustered abundantly on the axons adjacent to VMs in the co-culture treated with GDNF or NGF. Bars indicate 20 µm. **B)** The fraction of synaptic vesicles (SynI-positive area) on VMs (GFP-positive area). Values are means±SD of 5 co-cultures in each group. *Significantly different from control at p<0.05. #Significantly different from co-cultures treated with NGF at p<0.05 **C, D)** Images were obtained by transmission electronmicroscopy in proximity co-cultures treated for 5 days either with GDNF (10 ng/ml) (C) or NGF (50 ng/ml) (D). Nerve terminals (NT) contain granular vesicles (arrows), multi-vesicular bodies (arrow heads). These terminals closely appose to the plasma membrane of the VMs(*).Mitochondria (Mit) and myofibrils (Mf) are abundant in the cytoplasm of the VMs. Z: Z-lines. Bars indicate 500 nm.

**Figure 3:**
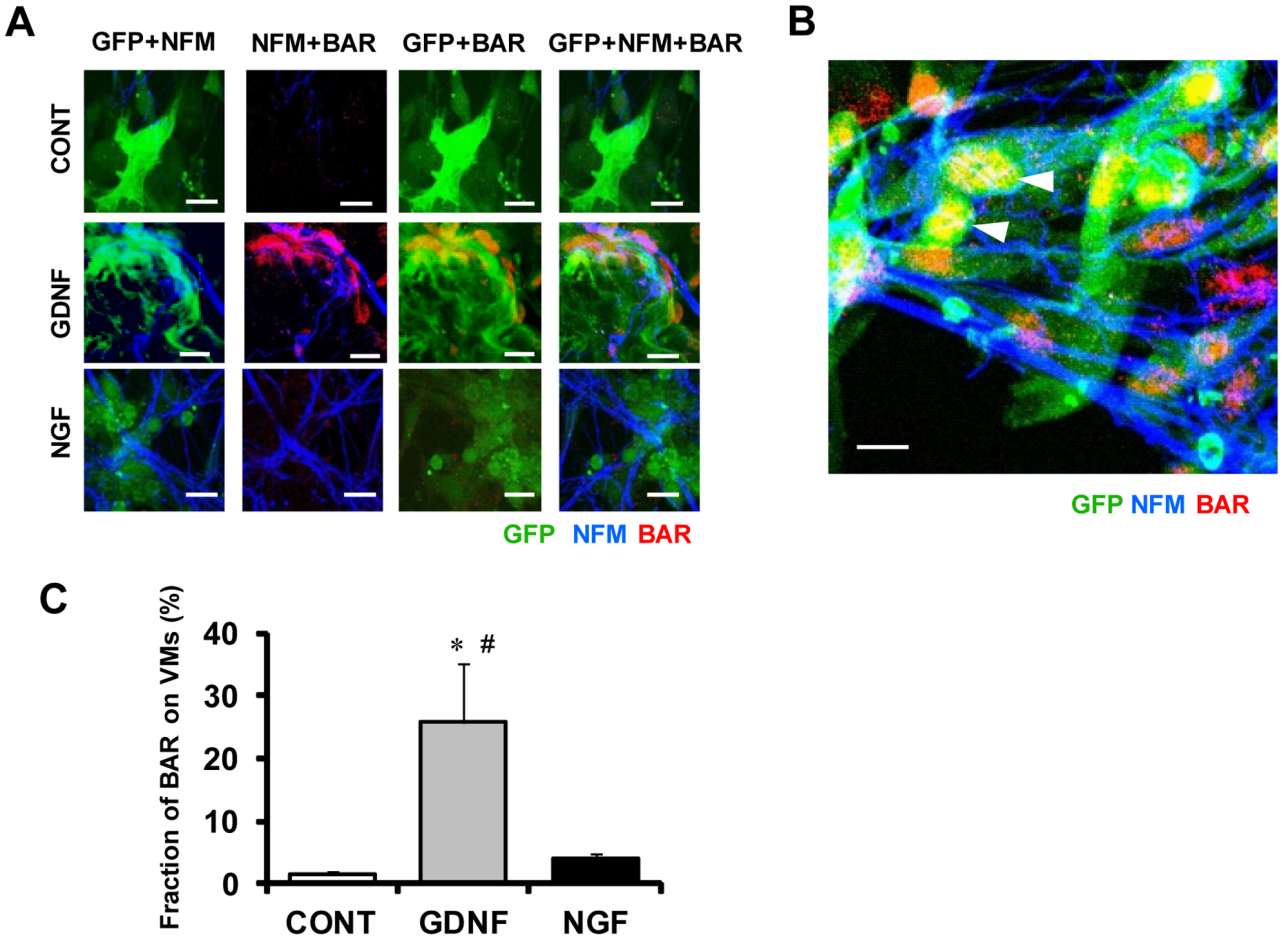
Expression of β1-adrenergic receptors in sympathetic neurons adjacent to VMs. Proximity co-cultures of GFP-knocked-in VMs and SNs (5 days) were immunolabeled for neurofilament-M (NFM, blue) and β1-adrenergic receptors (BAR; red). **A)** Representative fluorescence images of co-cultures treated with vehicle only were used as controls (CONT, top), treated with GDNF 10 ng/ml (middle) and treated with NGF 50 ng/ml (bottom). Pictures from left to right are labeled for GFP+NFM, NFM+BAR, GFP+BAR, and GFP+NFM+BAR. The immunopositive domains for BAR, which are most abundant with GDNF, are recognized on the surface membrane of VMs in contact with axons from SNs; the overlapping is colored by orange (GFP+BAR) or purple (NFM+BAR). **B)** Treatment with GDNF in co-cultures also leads to nuclear or perinuclear expression of BAR in VMs (white arrow heads). **C)** The density of immunopositive BAR distributing on VMs (GFP-positive area). Values are means+SD of 4 co-cultures in each group. *Significantly different from control at p<0.05. #Significantly different from co-cultures treated with NGF at p<0.05. Bars indicate 20 µm.

**Figure 4:**
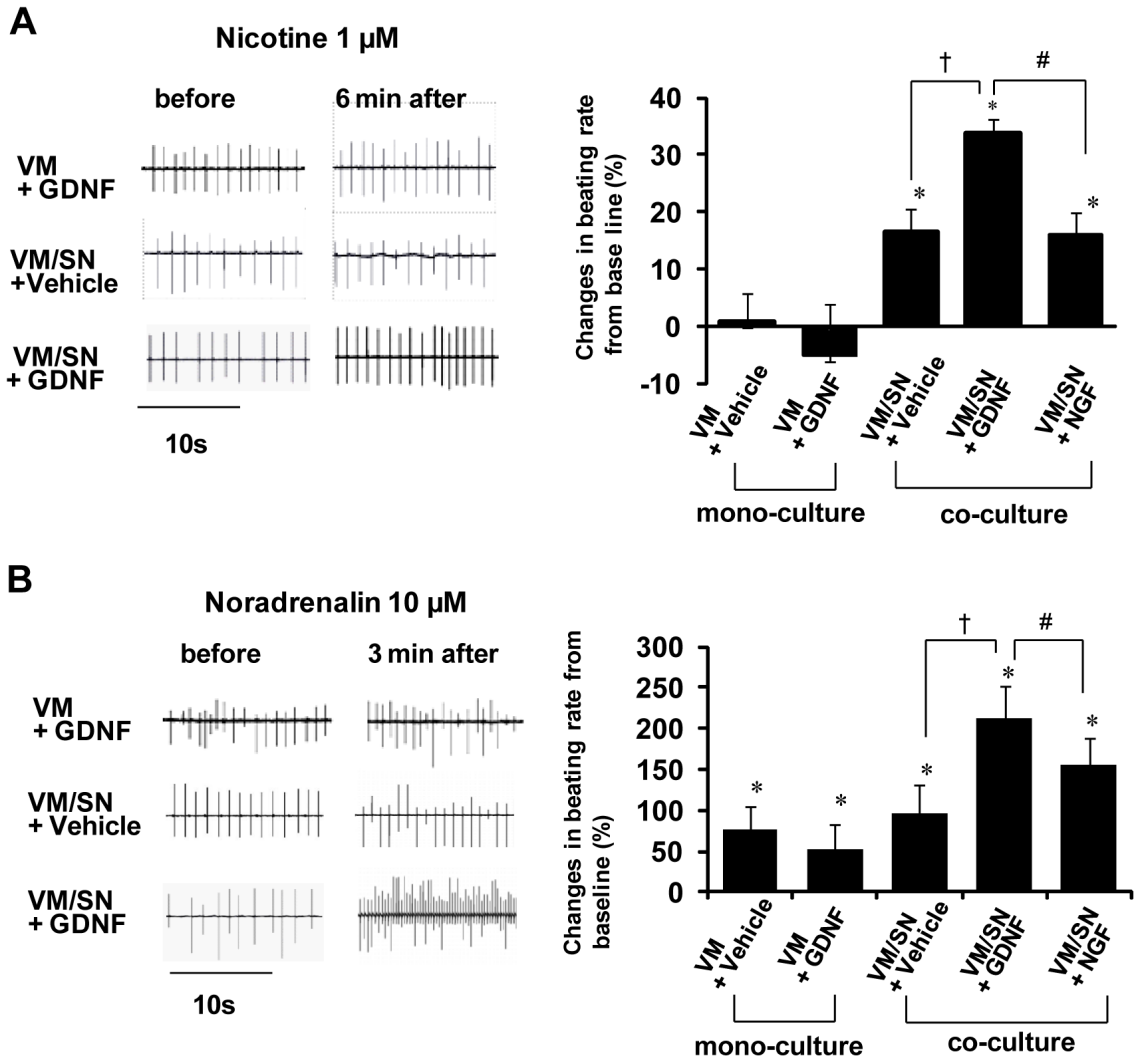
Pre- and post-synaptic stimulation by nicotine and noradrenalin on the spontaneous beating of cardiomyocytes. **A)** Effects of nicotine (1 µM). Left: representative recording of extracellular potentials before and 6 min after application of nicotine to a mono-culture of VMs treated for 5 days with GDNF 10 ng/ml ([VM+GDNF]), co-cultures of VMs/SNs treated with vehicle only ([VM/SN+Vehicle], and co-cultures treated 5 days with GDNF 10 ng/ml ([VM/SN+GDNF]). Right: percent changes of spontaneous beating rate from baseline. Values are means±SD (n = 4), *p<0.05 vs. VM+GDNF. **B)** Effects of noradrenalin (10 µM). Left: representative extracellular potentials of VMs before and 3 min after application of noradrenalin to a mono-culture of VMs/SNs treated for 5 days with GDNF 10 ng/ml ([VM+GDNF]), co-cultures of VMs/SNs treated with vehicle only ([VM/SN+Vehicle]), co-cultures treated 5 days with GDNF 10 ng/ml ([VM/SN+GDNF]). Right: percent changes of spontaneous beating rate from baseline. [VM+Vehicle] : mono-cultures of VMs treated with vehicle only, [VM+NGF]: mono-cultures of VMs treated 5 days with NGF 50 ng/ml, [VM+GDNF]: mono-cultures of VMs treated 5 days with GDNF 10 ng/ml, respectively. Values are means±SD (n = 4). *Significantly different from baseline in each group (p<0.05, n = 4); †p<0.05 vs the VM/SN group (n = 4); #p<0.05 vs the VM/SN+NGF group (n = 4).

VMs and SNs of the superior cervical ganglion were obtained from wild-type or green fluorescent protein (GFP)-transgenic 1-day-old neonatal Wistar rats through enzymatic digestion[14]–[17]. In the present study, we newly developed a proximity co-culture system (Figure 1A) which is different from a random co-culture system used in our previous study [13]. The dissociated SNs and VMs were used for proximity co-culture: SNs (4×10^5^/ml) and the VMs (2×10^6^/ml) were seeded separately on a gelatin-coated cover slip or 64-electrode array (MED-P545A: Alpha MED Scientific Inc, Ibaraki, Japan) at a close distance (1 mm) to observe the outgrowth of axons toward VMs. A glass ring frame (outer diameter 6 mm, inner diameter 4 mm) was placed initially (15 hours) for their separation (VMs inside and SNs outside). (Figure 1–4)

SNs/VMs were cultured in Dulbecco's Modified Eagle's Medium (Sigma-Aldrich, St. Louis, MO) containing 10% (vol/vol) fetal bovine serum (GibcoBRL, Gaithersburg, MD), insulin, transferrin, selenite liquid media supplement (Sigma-Aldrich) and cytosine arabinofuranosid (1 µmol/L: Sigma-Aldrich), at 37°C in an incubator with 5% CO_2_ and 98% humidity. The culture medium was supplemented either with NGF 50 ng/ml (Sigma-Aldrich), GDNF 10 ng/ml (R&D systems). Cell cultures were continued for 5 days to observe the axon outgrowth of SNs towards the VMs.

### *In vivo* Rat Model of Cardiac Denervation

8 weeks-old male Wistar rats were anesthetized with pentobarbital (50 mg/kg) and mechanically ventilated. After midline excision, the heart was exposed and a ring-shaped cryo-injury was induced in epicardial surface of the left ventricular free wall with a glass ring (outer diameter 6 mm, inner diameter 4 mm) frozen by liquid nitrogen. Then, the rats were divided into two groups: (1) GDNF and (2) Control groups. In the GDNF group, PBS containing recombinant adenoviruses encoding GDNF (AdGDNF: 2x10^6^ viral particles) was injected into the inner area of the injured ring. In the control group, phosphate-buffered saline (PBS) containing recombinant adenoviruses encoding GFP (AdGFP) was injected into the inner area of the injured ring. After the injection, chest walls were closed and rats were transferred to animal cages to recover from anesthesia. Five days later, the rats were anesthetized with pentobarbital. Then, the hearts were extracted and fixed for the consequent immunohistochemistry to examine the axon growth to the cryoinjured area. Whole-mount immunolabeling for NFM was carried out with the use of 3,3′-diaminobenzidine, tetrahydrochloride (DAB) as the secondary antibody.

### Immunofluorescence Labeling

Cultured cells were fixed with PBS containing 2% paraformaldehyde for 15 min at 4°C and permeabilized with 0.05% Triton X-100, then washed twice with PBS and blocked in PBS containing 10% bovine serum albumin for 1 hour at room temperature. Cells were incubated overnight at 4°C with primary antibodies: anti-α-actinin (AA) antibody (mouse monoclonal 1∶200; Sigma-Aldrich), anti-neurofilament M (NFM) (rabbit polyclonal or mouse monoclonal; 1∶200; Chemicon International, Temecula, CA), anti-synapsin I (SynI) (rabbit polyclonal; 1∶200; Chemicon International), anti-β1-adrenergic receptors (BAR) antibody (rabbit polyclonal; 1∶200; Affinity BioReagents, Rockford, IL), anti-GDNF antibody (goat polyclonal; 1∶200; R&D systems, Minneapolis, MN). The samples were then incubated for 1 hour at room temperature with a 1∶200 (v/v) dilution of appropriate secondary antibodies: Alexa Fluor 488-conjugated goat anti-mouse immunoglobulin G (IgG) (1∶200; Invitrogen, Carlsbad, CA), Alexa Fluor 568-conjugated goat anti-rabbit IgG (1∶200; Invitrogen), and Alexa Fluor 633-conjugated donkey anti-goat IgG (1∶200; Invitrogen). Immunofluorescence images were acquired using a confocal laser-scanning microscope (LSM510; Carl Zeiss MicroImaging, Inc., Jena, Germany). The fractions of NFM-positive axon, SynI and BAR over VMs were calculated using Image-pro Plus software (MediaCybernetics,Inc., Bethesda, MD). The hearts excised from adult rats after cryodenervation were immunolabeled similarly for NFM, AA and GDNF.

### Electron Microscopy

Co-cultures of CMs/SNs were fixed with 2.5% glutaraldehyde and 2% paraformaldehyde in 0.1M phosphate buffer for 15 min, and then post-fixed with 1.0% OsO_4_ in 0.1M phosphate buffer containing 4.5% sucrose for 1 hour. They were embedded with epoxy resin. Ultra-thin sections were prepared and examined with a JEPL1210 electron microscope (JEOL Ltd, Akishima, Japan). The images were taken at 10,000 power and scanned into a computer (at 300 dpi by a Dimage scan multi PRO, MINOLTA, Japan).

### Electrophysiological Study

Spontaneous beating activity of VMs co-cultured with SNs was assessed by recording extracellular potentials of VMs by using a 64-electrode array system (MED64 system; Alpha MED Scientific Inc, Ibaraki, Japan). To evaluate the presynaptic functional coupling, 1 µM nicotine ([-]-nicotine hydrogen tartrate salt; Sigma-Aldrich) was added to stimulate SNs [18]. To evaluate the postsynaptic function via β_1_-adrenergic receptors (BAR), noradrenaline (10 µM; Daiichi-Sankyo, Japan) was used as an agonist. Average spontaneous beating rates of VMs during each 3 min were obtained.

### Overexpression of GDNF in VMs using Recombinant Adenovirus

Recombinant adenoviruses encoding GDNF (AdGDNF) were prepared as described previously [19] VMs were infected with AdGDNF at a multiplicity of infection (m.o.i) of 10. The AdGDNF expressing VMs were plated in the proximity with SNs and cultured without the supplementation of GDNF. Mock-transfected CMs were used as a control.

### Cultures of Induced-pluripotent stem (iPS) Cells

Detailed information for experimental procedures for the culture of iPS cells has been shown in Text S1 [20].

## Results

### Effects of NGF and GDNF on Sympathetic Axon Growth Toward VMs

We examined the effects of GDNF and NGF on the axon outgrowth toward VMs using the proximity co-culture system (Figure 1). Time-lapsed microscopic imaging demonstrated that axons grew toward VMs as if the axons were searching for “target cells” (Figure 1B) (Sequential images are also available in Video S1). Figure 1B shows representative pictures of immunolabeling for neurofilament-M (NFM, neural marker, red) and α-actinin (AA, cardiac marker, green). The projection of axon toward VMs in the presence of GDNF (10 ng/ml) or NGF (50 ng/ml) was much more abundant compared to control. Figure 1D summarizes the results obtained in 10 culture dishes in each group. The fraction of axon (NFM-positive) covering the total area of VMs (AA-positive) in the culture treated with GDNF was increased by 9-fold compared to control (p<0.01), whereas this increase was 4-fold with NGF (p<0.01). Also, the increase with GDNF was significantly larger than that with NGF (p<0.05). These observations reveal that GDNF is more potent than NGF in stimulating axon growth toward VMs. In monocultures of sympathetic neurons, axon growth was enhanced with both GDNF and NGF compared to control, but no substantial difference was observed between the two neurotrophic factors (Figure 1E).

### Synapse Formation between VMs and SNs

We next investigated synapse formation between sympathetic axons and VMs in the proximity co-cultures of GFP-knocked-in VMs and SNs (Figure 2). Figure 2A shows a typical example of an experiment where SNs and synaptic vesicles were labeled by anti-neurofilament-M (NFM, blue) and anti-synapsin I (SynI, red) antibodies, respectively. SynI is a peripheral membrane protein of synaptic vesicles [21]. The synaptic vesicles (red punctuate domains) were abundant in the co-cultures treated with either GDNF or NGF; they were clustered on the axons (blue) adjacent to VMs (green). The increase of synaptic vesicles with GDNF was more prominent than that with NGF. Figure 2B summarizes the density of SynI-positive area superimposed on the GFP-positive area (VMs). Averaged values with GDNF and those with NGF were both significantly larger than control (n = 5, p<0.05). The increase with GDNF was much more prominent than that with NGF (p<0.05). These results show that GDNF not only stimulates the axon growth of SNs but also facilitates the presynaptic formation of SNs that have made contact with VMs.

The ultrastructure of the contact area between nerve terminals (from SNs) and VMs was examined by electron microscopy. Figure 2C shows a representative image of co-culture supplemented with GDNF (10 ng/ml). The nerve terminals were large in size and contained granular vesicles and multi-vesicle bodies. Figure 2D shows nerve terminals with granular vesicles close to the opposing sarcolemma of VMs in a section from a co-culture with NGF (50 ng/ml).

### Expression of β_1_-adrenergic Receptors (BARs) on VMs

Catecholamines (norepinephrine and epinephrine) are the major neurotransmitters released from SNs acting primarily on β_1_-adrenergic receptors (BARs) in VMs [22]. We examined the effects of GDNF and NGF on the expression of BARs in proximity co-cultures of GFP-expressing VMs and SNs (Figure 3). Figure 3A shows representative immunolabeling pictures, where BARs on VMs and axons from SNs were labeled by anti-BAR and anti-NFM antibodies, respectively. In control, BARs (red spots) were detected only scarcely. In a culture treated with GDNF or NGF, the expression of BARs was increased. In a culture treated with GDNF, BARs were detected most abundantly. Figure 3B shows that the immunopositive domains for BARs were not only clustered at the sarcolemma, but were also observed in the nuclear or perinuclear region of VMs, which were in contact with axons from SNs (Figure 3B). Figure 3C summarizes the data obtained from 4 culture dishes in each group. The density of immunopositive BARs over the entire area of VMs in the cultures treated with GDNF was significantly higher than those with NGF and control (p<0.05).

### Functional Synaptic Coupling between SNs and VMs

We examined the effects of presynaptic and postsynaptic stimulation by nicotine and noradrenaline, respectively, in the absence and presence of GDNF or NGF to assess functional coupling between SNs and VMs. Figure 4A (right panels) shows the effects of nicotine (1 µM) applied to mono-cultures of VMs, mono-cultures of VMs treated with GDNF 10 ng/ml ([VM+GDNF]), co-cultures of VMs/SNs without treatment ([VM/SN]), co-cultures of VMs/SNs treated with GDNF 10 ng/ml ([VM/SN+GDNF]), VMs/SNs treated with NGF 50 ng/ml ([VM/SN+NGF]). Nicotine had no effect on the spontaneous beating rate of mono-cultures of VMs despite treatment with GDNF ([VM+GDNF]). In contrast, nicotine increased the spontaneous beating rate in all co-cultures of VMs/SNs. Therefore, SNs are required for the effect of nicotine. The positive chronotropic effect developed slowly and took ∼6 min to reach a quasi steady-state. The nicotine-induced increase of spontaneous beating rate from baseline in VM/SN+GDNF was significantly larger than that in VM/SN+NGF. The left panels of Fig. 4A show selected electrograms in the most relevant 3 of the 5 groups.

Figure 4B (right panels) shows the effects of noradrenaline (10 µM) applied to mono-cultures of VMs treated with vehicle only ([VM+vehicle]), GDNF 10 ng/ml ([VM+GDNF]), co-cultures of VMs/SNs with vehicle only ([VM/SN+vehicle]), NGF 50 ng/ml (VM/SN+NGF), and GDNF 10 ng/ml ([VM/SN+GDNF]). Noradrenaline increased the spontaneous beating rate of VMs significantly in all 5 experimental groups after 3 min (p<0.05). The increase in beating rate was larger (p<0.05) in the co-cultures with GDNF compared with the co-cultures without neurotrophic factors (VM/SN). Finally, the increase in beating rate was also larger (p<0.05) in the co-cultures with GDNF (VM/SN+GDNF) than in the co-cultures with NGF (VM/SN+NGF).

The left panels of Figure. 4B show selected electrograms in the most relevant 3 of the 5 groups.

### GDNF-induced Axon Guidance *in vitro*

**Figure 5:**
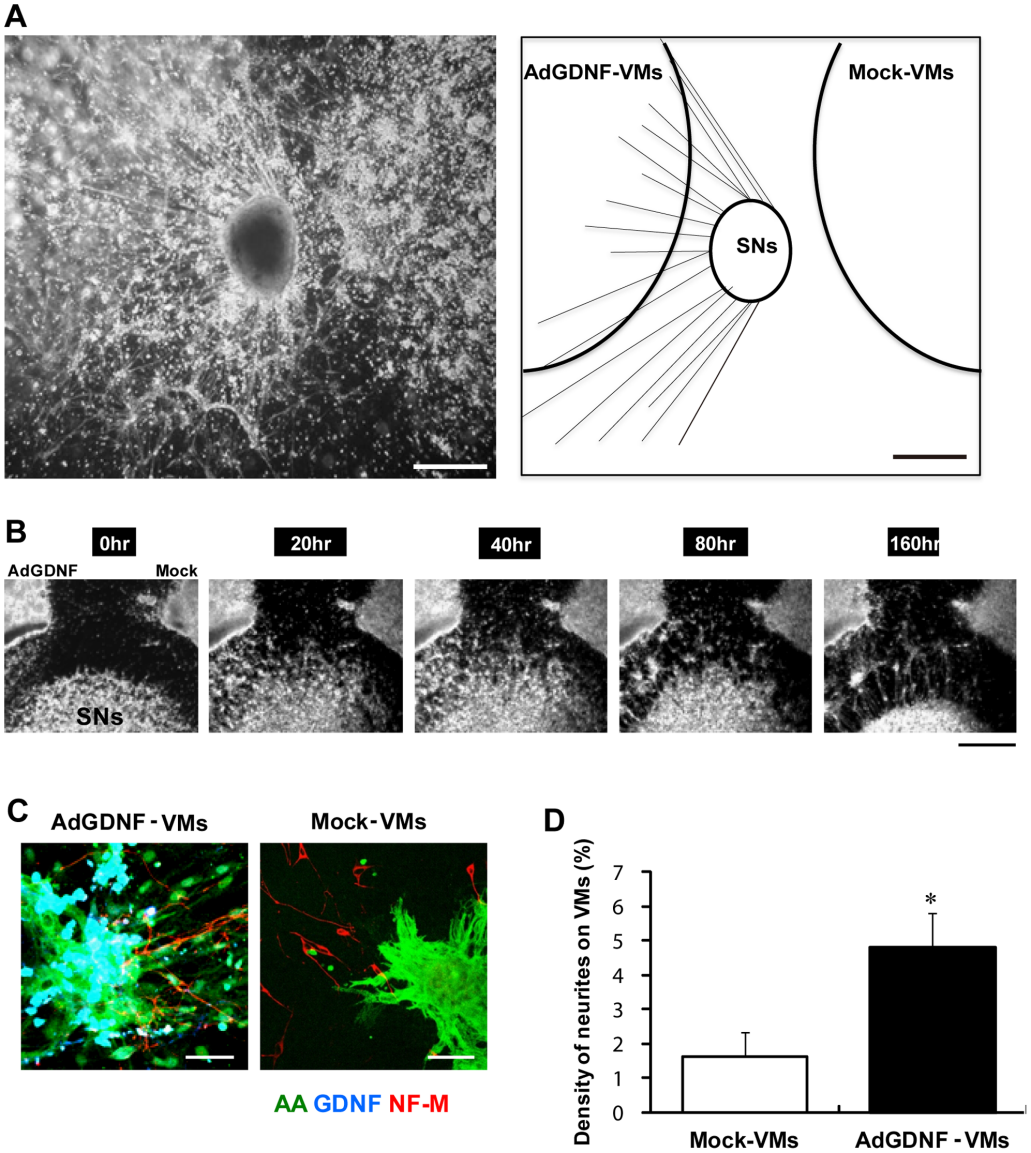
Axon growth of SNs toward GDNF-expressing VMs. GDNF- or mock-transfected VMs were prepared by using adenovirus vectors (AdGDNF-VMs and Mock-VMs) and co-cultured with SNs at a close distance (∼1 mm). **A)** A bright field image of the triangular co-culture (day 5) (left) and its schematic illustration (right). The axons from SN grow predominantly toward AdGDNF-VMs. Bar indicate 1 mm. Video images (Video S2) are available online. **B)** Sequential time-lapsed images. The pictures were taken at 0, 20, 40, 80 and 160 hours after removal of the glass-ring separation. Bar indicate 1 mm. **C)** Representative immunolabeling images of neurofilament-M (NF-M, red), α-actinine (AA, green) and GDNF (blue). Left: AdGDNF-VMs close to SNs, Right: Mock-VMs close to SNs. The NFM-positive axons (red fibrous structure) distribute abundantly on AdGDNF-VMs (green+blue), whereas only scarcely on Mock-VMs (green). Bars indicate 20 µm. **D)** The fraction of axon (NFM-positive area) distributing on VMs (AA-positive area). Values are means±SD of 5 co-cultures in each group. *significantly different from mock-VMs.

Our results show that exogenously-applied GDNF has a potent effect on cardiac sympathetic innervation. It is conceivable to assume that endogenous GDNF released from VMs has a similar effect. To address this issue, we prepared VMs overexpressing GDNF by using adenoviral vectors. Co-cultures of AdGDNF-VMs and mock-transfected VMs (Mock-VMs) with SNs in close proximity (1 mm) are shown (Figure 5). When the glass frame separating VMs (outside) and SNs (inside) was removed, the axons from SNs grew much more prominently toward AdGDNF-VMs, compared with Mock-VMs. Figure 5A shows a representative picture on day 5. Figure 5B shows sequential time-lapse microscope images from 0 to 160 hours. The axons grew as if they were searching for AdGDNF-VMs (A movie is available in Video S2).

The axons growth toward the two different groups of VMs was quantified by immunolabeling. The axons were labeled by anti-neurofilament-M antibody (red), whereas cardiomyocytes and GDNF were labeled by anti-α-actinin and anti-GDNF antibodies, respectively (green and blue). Representative fluorescence images are shown in Figure 5C. The axons recognized on AdGDNF-VMs were much more abundant than those on Mock-VMs. The results obtained from 5 dishes are summarized in Figure 5D. The percentage of axons covering AdGDNF-VMs was significantly larger than that on Mock-VMs.

Effects of GDNF expression on axon guidance on iPS cell-derived cardiomyocytes (iPSCMs) were also examined using close proximity (1 mm) co-cultures of AdGDNF-iPSCMs and mock-transfected iPSCMs (Mock-iPSCMs) with SNs. As a result, AdGDNF-iPSCMs attracted significantly more abundant axons than Mock-iPSCMs. (Figure S2 and Video S3).

### *In vivo* Adult Rat Heart Model of Denervation and GDNF Overexpression

**Figure 6:**
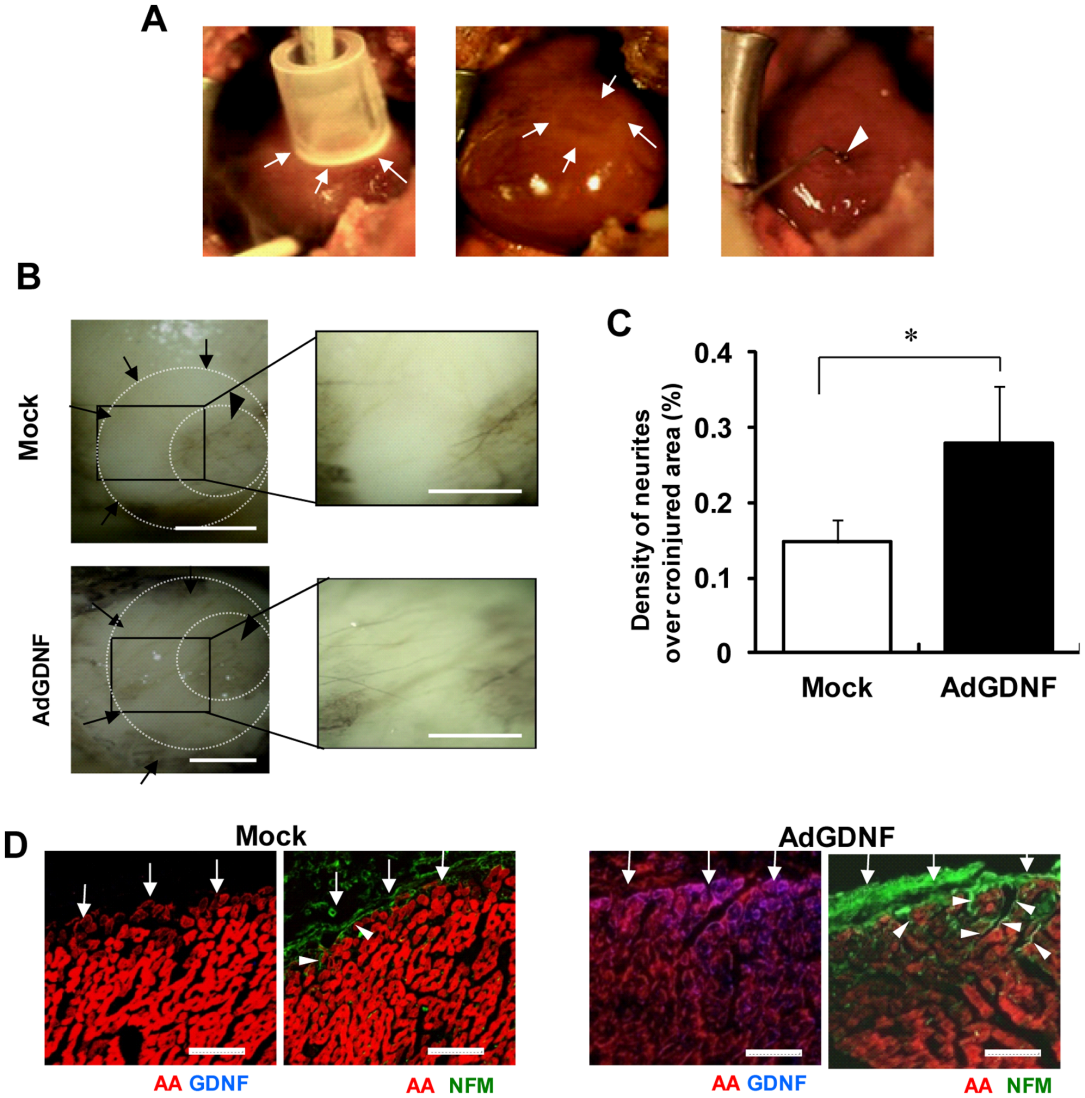
Re-innervation in a rat model of denervation by cryoinjury. **A)** Induction of denervation in rat hearts *in vivo*. A ring-shaped denervation was induced by cryoinjury on the epicardial surface of the left ventricular free wall (arrows in left and middle panels). Ad-GDNF was injected into the inner area of the injured ring (arrowhead in right). Ad-GFP was injected in controls. **B)** Representative pictures of axon growth in the ring-shaped denervated area 5 days after the creation of cryoinjury. The hearts were isolated and whole-mount labeled for neurofilament-M (NFM, DAB stain). In the control heart (top), axons were detected only sparsely in the denervated area (arrows). In the Ad-GDNF-injected heart (bottom), in contrast, axons grew abundantly across the denervated area (arrows). Arrowheads indicate the uninjured central area (inner ring). Right panels show magnified images of the rectangular zones (injured areas). Bars indicate 1 mm. **C)** The percentage of the denervated area covered by axons (NFM-positive). Values are means±SD. *p<0.05 vs control (n = 5). **D)** Representative immunolabeling images of NFM (green), α-actinine (AA, red) and GDNF (blue) in the sections across the long axis of the rat hearts (denervated area). Note that GDNF was abundantly expressed in the myocardium, and the NFM-positive axons grew from the epicardial surface (arrows) into the midmyocardium in the AdGDNF heart (bottom). In the control heart (top), in contrast, GDNF was undetectable in the myocardium and the axons were recognized only scarcely on the epicardial surface. Bars indicate 50 µm.

We examined the effects of GDNF on the sympathetic re-innervation in the injured myocardium in an *in vivo* model of adult rats in order to obtain more insight into its pathophysiological role. A ring-shaped epicardial surface of the left ventricular free wall was cryoinjured (Figure 6A: left) to cause denervation (Figure 6A middle). Then, AdGDNF was injected into the inner area of the injured ring (Figure 6A: right). Figure 6B shows representative pictures of whole-mount immunohistochemistry in the control (top) and the AdGDNF-treated hearts (bottom) 5 days after the creation of cryoinjury. In the control heart, axons were sparsely detected in the denervated area (arrows). In contrast, appreciable axon growth (reinnervation) across the denervated area (arrows) was recognized in the AdGDNF-injected heart. The fraction of axons recognized in the denervated area in the AdGDNF-injected group was significantly larger than that in the control group (Figure 6C). Figure 6D shows representative images of immunolabeling for AA and GDNF (left) and for AA and NFM (right) in sections across the long axis of the heart. In the AdGDNF-injected heart, GDNF was expressed abundantly in the myocardium, and NFM-positive axons grew from the epicardial surface toward the midmyocardium. In the control heart, in contrast, GDNF was undetectable and only small numbers of NFM-positive axons were recognized at the epicardial surface, but not invading the deeper layers.

## Discussion

In the present study, we showed that exogenously applied GDNF promotes sympathetic axon growth toward both native and iPS cell-derived cardiomyocytes, more potently than NGF, with morphological and functional synapse formation with concomitant increase of ß_1_-adrenergic receptors in cardiomyocytes. The facilitating effect of GDNF was also demonstrated in the *in vivo* rat model of myocardial denervation. The results suggest that GDNF acts as a potent chemoattractant for axon guidance of SNs both *in vitro* and *in vivo*.

### GDNF Promotes Axon Growth Toward Cardiomyocytes more Potently than NGF

After myocardial injury, cardiac autonomic nerves undergo Wallerian degeneration, which is followed by neurolemma cell proliferation and axonal regeneration, leading to re-innervation. Various neurotrophic factors have been suggested to be involved in this regenerative process, including NGF, BDNF, GDNF, and artemin. Among these neurotrophic factors, NGF has been studied most extensively [6]–[8]. NGF released from ischemic cardiac tissue plays a pivotal role in sympathetic nerve sprouting, which may cause life-threatening ventricular arrhythmias [23]; [24]. On the other hand, a role of GDNF in cardiac autonomic (re)innervation has received little attention because of relatively low mRNA and protein expression levels in adult cardiac tissue [25]. Previously, we have shown that GDNF most potently promoted axon growth from SNs to VMs using random co-cultures of VMs and SNs among neurotrophic factors [13]. In addition, we have confirmed that mRNA and protein of GDNF are expressed in rat hearts at embryonic and neonatal stages, although the expression is below the detectable level in adulthood [13]. In the present study, a newly developed proximity co-culture system of VMs and SNs also revealed that GDNF toward VMs, more clearly compared to NGF. It has been reported that GDNF acts through Ret signals [26]. Our previous report demonstrated that GDNF may act directly on SNs through Ret signals, but not by indirect effect through NGF, since GDNF did not increase NGF levels [13]. We also showed that even under the addition of anti-NGF antibody, the sympathotrophic effect of GDNF was preserved even under the NGF-depleted condition induced by the addition of anti-NGF antibody [13].

### GDNF Induces Functional Synapse Formation between Sympathetic Neurons and Cardiomyocytes

Sympathetic axons and cardiac myocytes are coupled morphologically and functionally by synapse formation. It was recently demonstrated in co-cultures of sympathetic ganglionic neurons (SGNs) and cardiomyocytes that sympathetic innervation and synaptogenesis influence the structure of the sarcolemmal membrane and the organization and distribution of β_1_ and β_2_ adrenergic receptors [18]. Conversely, cardiac myocytes induce presynaptic differentiation in contacting axons and synaptic vesicle accumulation at the sites of contact [18]. In the present study, we have shown that exogenous application of either GDNF (10 ng/ml) or NGF (50 ng/ml) increases the expression of synapsin I, a peripheral membrane protein of synaptic vesicles and the expression of β_1_ adrenergic receptors in VMs at their junction with axons. The upregulation of SynI and BARs induced by GDNF was 4–7 times more potent than that induced by NGF. These observations suggest that both GDNF and NGF enhance not only axon growth but also synaptogenesis, and -again- the enhancement with GDNF is much stronger than that with NGF. Interestingly, pronounced upregulation of BARs in VMs with GDNF was recognized not only at the sarcolemma but also in the nuclear or perinuclear regions. Immuno-reactive β_1_ and β_3_ adrenoceptors at the nuclear membranes were recently demonstrated in murine ventricular cardiomyocytes [22]. The pathophysiological significance of nuclear or perinuclear expression of BARs is terra incognita. But it suggests, of course, a role in transcription and thereby a role in remodeling. Our morphological and molecular data all had a functional counterpart, both at the presynaptic and postsynaptic level. Chronotropic responses to nicotine and noradrenaline were stronger in the presence of GDNF compared with NGF and the absence of neurotrophic factors.

### Axon Guidance by GDNF *in vitro*

Our experiments using exogenously-applied GDNF suggested that this molecule acts as a potent chemoattractant if it were excreted by VMs. To verify this assumption, we created VMs and iPSCMs overexpressing GDNF by adenovirus infection (AdGDNF-VMs, iPSCMs). In the proximity co-culture experiments separately placed AdGDNF-VMs, Mock-VMs and SNs, axon growth from SNs indeed occurs predominantly toward AdGDNF-CMs, confirming the above hypothesis. Previously, Martinelli *et al.* demonstrated that in a rat model of chemical sympathectomy by 6-hydroxydopamine, the GDNF protein level increased transiently prior to axonal regrowth in a cardiac tissue homogenate [9]. The same group reported that in rats infected with *Trypanosoma cruzi* (Chagas disease) anti-GDNF gold particles in atrial granules increased transiently at the time of maximal autonomic denervation [10]. In contrast, in a mouse model of myocardial infarction and subsequent sympathetic nerve sprouting, myocardial gene expression of GDNF (estimated by RT-PCR)) was, unlike NGF, virtually unchanged [3]. In rats, cardiac expression of GDNF is limited to the embryonic and early post-natal stage [13], whereas NGF is abundantly expressed in the human adult heart [25]. Maltinellei et al. showed increased GDNF mRNA expression in the endoplasmic reticulum of rat cardiomyocytes in response to chemical sympathetctomy. They also confirmed GDNF protein expression in granules (atrial myocytes) and cytoplasm (ventricular myocytes), suggesting that GDNF may be secreted from cardiomyocytes [9]. Nevertheless, we cannot eliminate other possible cell sources of GDNF secretion from the heart, because artemin, another member of the GDNF family, has been shown to be secreted from vascular smooth muscle [12].

### *In vivo* Adult Rat Heart Model of Denervation

In our *in vivo* adult rat heart model of denervation created by cryoinjury, no substantial GDNF upregulation was recognized in the denervated regions of the control hearts (without AdGDNF injection), and this was associated with moderate axon growth toward the denervated region. In contrast, in the rat hearts with AdGDNF injection inside the denervated region considerable GDNF expression and prominent axon growth was observed in the denervated region. These results do not support a role for endogenous GDNF *per se* in the reinnervation of adult heart following injury, but they do suggest that GDNF can potentially be used to promote reinnervation.

Myocardial regeneration therapy by transplantation of stem cell-derived cardiac tissues attracts a great deal of attention as an innovation for the treatment of heart failure refractory to conventional therapies. To obtain functional regenerated myocardium *in vivo*, appropriate autonomic reinnervation will be required for the transplanted cardiac myocytes to function in response to a variety of physical demands. Our experiments using proximity co-cultures of iPS-derived CMs overexpressing GDNF (AdGDNF-iPSCMs), mock-transfected iPSCMs and SNs have revealed that GDNF was expressed in iPSCMs and certainly acts a potent chemoattractant for the sympathetic axon guidance.

### Physiological and Therapeutic Implication

The present study demonstrated that GDNF promotes sympathetic innervation in both native cardiac cells and stem cell-derived cardiac cells. The results implicated that local delivery of GDNF would exert strong effects to navigate sympathetic axons to target cardiac tissues in cases of myocardial injury or transplantation of regenerated myocardial tissue.

## Supporting Information

Figure S1 **iPS cell-derived cardiomyocytes (iPSCMs). A)** A representative image of a spontaneous beating mass of iPSCMs microdissected from iPS cell-derived EBs. **B)** Extracellular potentials recorded in spontaneous beating mass shown in (A). Each trace was obtained from corresponding electrodes shown in (A). Potentials were recorded with MED64 system. **C,D)** Effects of isoproterenol (1 µM) (C) and carbamyl choline (10 µM) (D) on iPSCMs. Values are means±SD. *p<0.05 vs baseline. **E)** Representative immunolabeling images of nanog (green) and actin (red) in spontaneous beating iPSCMs. Note most of the cells are positive for actin. Bar indicate: 50 µm.
(EPS)

Figure S2 **Axon growth of sympathetic neurons toward GDNF-expressing iPSCMs.** GDNF- or mock-transfected iPSCMs were prepared by using adenovirus vectors (AdGDNF-iPSCMs and Mock-iPSCMs) and co-cultured with SNs at a close distance (∼1 mm). **A)** A bright field image of the triangular co-culture (day 5) (left) and its schematic illustration (right). The axons from SN grow predominantly toward AdGDNF-iPSCMs. Bar indicate 1 mm. Video images (video S3) are available online. **B)** Sequential time-lapsed images. The pictures were taken at 0, 20, 40, 80 and 140 hours after removal of the glass-ring separation. Bar indicate 1 mm. **C)** Representative immunolabeling images of neurofilament-M (NF-M, blue), α-actinine (AA, red) and nanog-GFP (green). Left: AdGDNF-iPSCMs close to SNs, Right: Mock-iPSCMs close to SNs. The NFM-positive axons (blue fibrous structure) distribute abundantly on AdGDNF-iPSCMs, whereas only scarcely on Mock-iPSCMs. Bars indicate 20 µm. **D)** The fraction of axon (NFM-positive area) distributing on iPSCMs (AA-positive area). Values are means±SD of 5 co-cultures in each group. *p<0.05 vs mock-iPSCMs.
(EPS)

Text S1 **Cultures of induced-pluripotent stem (iPS) cells.** A cell line of Nanog-GFP knock-in iPS cells generated from Fbx15 iPS cells were provided by Dr. Shinya Yamanaka (Kyoto University) [20]. To maintain the iPS cells in undifferentiated state, the cells were grown on inactivated mouse embryonic fibroblasts (MEFs) with “undifferentiation maintaining medium” [DMEM (Sigma-Aldrich, St.Luis, MO) containing 15% (vol/vol) FBS (GibcoBRL, Gaithersburg, MD), and leukemia inhibitory factor (LIF) (10 mU/ml GibcoBRL) MEM non-essential amino acids (0.1mM, GibcoBRL) and 2-mercapto ethanol (100 µM, GibcoBRL)]. The iPS cells were then digested with trypsin-EDTA and suspended in the LIF-free differentiation medium [DMEM (Sigma-Aldrich, St.Luis, MO) containing 15% (vol/vol) FBS (GibcoBRL, Gaithersburg, MD), MEM non-essential amino acids (0.1mM, GibcoBRL) and 2-mercapto ethanol (100 µM, GibcoBRL)]. The iPS cells were cultured in small drops (each 20 µL containing 1,600–2000 cells) hanged from the lid of culture dish ("hanging-drop") to form spheroids (embryoid bodies: EBs) for 3 days. EBs were then transferred to tissue culture dishes (50 EBs per dish) and further cultivated for 7 days. Most EBs started showing spontaneous beating approximately 8 days after differentiation. Ten days after differentiation, spontaneously contracting EB outgrowths were microdissected under a microscope and plated on culture dishes with as described above. Microdissected clumps of beating cells were then infected with Ad-GDNF in the same protocol with neonatal VMs. Then, AdGDNF-expressing iPS-derived beating EBs and mock-transfected EBs were co-cultured adjacent to sympathetic neurons in the same culture dishes. Co-cultured iPS cells-derived EBs and SNs were fixed and subjected to immunofluorescence to examine the effect of GDNF on innervation to regenerated myocardium.
(DOCX)

Video S1 **Axon growth of sympathetic neurons toward cardiomyocytes.** Sequential time-lapsed microscopic images from 0 to 50 hours after the proximity (∼1 mm)co-cultures of SNs (right) and CMs (left) with the exogenous application of NGF (50 ng/ml).Note that axons from SNs are growing as if they are searching for “target” cells. See Figure 1 for details.
(MPG)

Video S2 **Axon guidance of sympathetic neurons by GDNF-overexpressing VMs.** Sequential time-lapsed microscopic images from 0 to 160 hours after the proximity (∼1 mm) co-cultures of SNs and AdGDNF-VMs (left upper) and Mock-VMs (right upper). Note axons from SNs are grown predominantly toward AdGDNF-VMs (left upper). See Figure 5 for details.
(AVI)

Video S3 **Axon guidance of sympathetic neurons by GDNF-overexpressing iPSCMs.** Sequential time-lapsed microscopic images from 0 to 144 hours after the proximity (∼1 mm) co-cultures of SNs and AdGDNF-iPSCMs (left upper) and Mock-iPSCMs (right upper). Note axons from SNs are grown predominantly toward AdGDNF-iPSCMs (left upper). See Figure S2 for details [20].
(WMV)
