# Supplementary material for: Axon Guidance of Sympathetic Neurons to Cardiomyocytes by Glial Cell Line-Derived Neurotrophic Factor (GDNF)
Source: PLoS One. 2013 Jul 3;8(7):e65202. doi: 10.1371/journal.pone.0065202 (PMC3701054; doi:10.1371/journal.pone.0065202)
Supplement: Text S1 — Cultures of induced-pluripotent stem (iPS) cells. A cell line of Nanog-GFP knock-in iPS cells generated from Fbx15 iPS cells were provided by Dr. Shinya Yamanaka (Kyoto University) [20]. To maintain the iPS cells in undifferentiated state, the cells were grown on inactivated mouse embryonic fibroblasts (MEFs) with “undifferentiation maintaining medium” [DMEM (Sigma-Aldrich, St.Luis, MO) containing 15% (vol/vol) FBS (GibcoBRL, Gaithersburg, MD), and leukemia inhibitory factor (LIF) (10 mU/ml GibcoBRL) MEM non-essential amino acids (0.1mM, GibcoBRL) and 2-mercapto ethanol (100 µM, GibcoBRL)]. The iPS cells were then digested with trypsin-EDTA and suspended in the LIF-free differentiation medium [DMEM (Sigma-Aldrich, St.Luis, MO) containing 15% (vol/vol) FBS (GibcoBRL, Gaithersburg, MD), MEM non-essential amino acids (0.1mM, GibcoBRL) and 2-mercapto ethanol (100 µM, GibcoBRL)]. The iPS cells were cultured in small drops (each 20 µL containing 1,600–2000 cells) hanged from the lid of culture dish ("hanging-drop") to form spheroids (embryoid bodies: EBs) for 3 days. EBs were then transferred to tissue culture dishes (50 EBs per dish) and further cultivated for 7 days. Most EBs started showing spontaneous beating approximately 8 days after differentiation. Ten days after differentiation, spontaneously contracting EB outgrowths were microdissected under a microscope and plated on culture dishes with as described above. Microdissected clumps of beating cells were then infected with Ad-GDNF in the same protocol with neonatal VMs. Then, AdGDNF-expressing iPS-derived beating EBs and mock-transfected EBs were co-cultured adjacent to sympathetic neurons in the same culture dishes. Co-cultured iPS cells-derived EBs and SNs were fixed and subjected to immunofluorescence to examine the effect of GDNF on innervation to regenerated myocardium. (DOCX) [file pone.0065202.s003.docx]

**Miwa et al.**

**Axon guidance of sympathetic neurons to cardiomyocytes by glial cell line-derived neurotrophic factor (GDNF)**

**SUPPORTING INFORMATION**

**Text S1**

**Cultures of induced-pluripotent stem (iPS) cells**

A cell line of Nanog-GFP knock-in iPS cells generated from Fbx15 iPS cells were provided by Dr. Shinya Yamanaka (Kyoto University) [20] . To maintain the iPS cells in undifferentiated state, the cells were grown on irradiated mouse embryonic fibroblasts (MEFs) with “undifferentiation maintaining medium” [DMEM (Sigma-Aldrich, St.Luis, MO) containing 15% (vol/vol) FBS (GibcoBRL, Gaithersburg, MD), and leukemia inhibitory factor (LIF) (10 mU/ml GibcoBRL) MEM non-essential amino acids (0.1mM, GibcoBRL) and 2-mercapto ethanol (100 μM , GibcoBRL)]. The iPS cells were then digested with trypsin-EDTA and suspended in the LIF-free differentiation medium [DMEM (Sigma-Aldrich, St.Luis, MO) containing 15% (vol/vol) FBS (GibcoBRL, Gaithersburg, MD), MEM non-essential amino acids (0.1mM, GibcoBRL) and 2-mercapto ethanol (100 μM , GibcoBRL)]. The iPS cells were cultured in small drops (each 20 µL containing 1,600-2000 cells) hanged from the lid of culture dish ("hanging-drop") to form spheroids (embryoid bodies: EBs) for 3 days. EBs were then transferred to tissue culture dishes (50 EBs per dish) and further cultivated for 7 days. Most EBs started showing spontaneous beating approximately 8 days after differentiation. Ten days after differentiation, spontaneously contracting EB outgrowths were microdissected under a microscope and plated on culture dishes with as described above. Microdissected clumps of beating cells were then infected with Ad-GDNF in the same protocol with neonatal VMs. Then, AdGDNF-expressing iPS-derived beating EBs and mock-transfected EBs were co-cultured adjacent to sympathetic neurons in the same culture dishes. Co-cultured iPS cells-derived EBs and SNs were fixed and subjected to immunofluorescence to examine the effect of GDNF on innervation to regenerated myocardium.
